# Coping with the New Era: Noise and Light Pollution, Hperactivity and Steroid Hormones. Towards an Evolutionary View of Bipolar Disorders

**DOI:** 10.2174/1745017901814010033

**Published:** 2018-02-28

**Authors:** MG Carta, A Preti, HS Akiskal

**Affiliations:** 1 Department of Health Sciences and Public Health, University of Cagliari, Cagliari, Italy; 2 University of California at San Diego USA

**Keywords:** Melatonin, Sleep, Biorhythms, Bipolar disorder, Quality of life, Globality

## Abstract

Human population is increasing in immense cities with millions of inhabitants, in which life is expected to run 24 hours a day for seven days a week (24/7). Noise and light pollution are the most reported consequences, with a profound impact on sleep patterns and circadian biorhythms. Disruption of sleep and biorhythms has severe consequences on many metabolic pathways. Suppression of melatonin incretion at night and the subsequent effect on DNA methylation may increase the risk of prostate and breast cancer. A negative impact of light pollution on neurosteroids may also affect mood. People who carry the genetic risk of bipolar disorder may be at greater risk of full-blown bipolar disorder because of the impact of noise and light pollution on sleep patterns and circadian biorhythms. However, living in cities may also offers opportunities and might be selective for people with hyperthymic temperament, who may find themselves advantaged by increased energy prompted by increased stimulation produced by life in big cities. This might result in the spreading of the genetic risk of bipolar disorder in the coming decades. In this perspective the burden of poor quality of life, increased disability adjusted life years and premature mortality due to the increases of mood disorders is the negative side of a phenomenon that in its globality also shows adaptive aspects. The new lifestyle also influences those who adapt and show behaviors, reactions and responses that might resemble the disorder, but are on the adaptive side.

## 1 CITIES NOISE AND LIGHT POLLUTION AND (MENTAL) HEALTH

Sleep is considered of critical importance in the onset, recurrence, dysfunction, and adverse health outcomes in bipolar disorder [1]. Sleep patterns are increasingly menaced by recent changes in the lifestyle of humans. Indeed, humans are increasingly amassing into enclaves of millions of inhabitants (big cities) in which life is expected to run 24 hours a day for seven days a week (24/7) [2]. Noise and light pollution by artificial lights are the most reported consequences of a 24/7 lifestyle, with profound impact on sleep pattern and circadian biorhythms.

Road traffic noise is associated with risk of psychiatric disorders, but poor sleep has been found to be a major co-determinant [3]. Thus, individuals with poor quality of sleep might be more vulnerable to the impact of road traffic noise on mental health even if it is difficult to understand the direction of causation. Indeed, is still undetermined whether those most affected by road traffic noise were already poor sleepers or poor sleep was the first manifestation of impairment due to road traffic, thus being first step in psychopathological decompensation.

The effect of artificial light on mental health is better known. Artificial light modifies daily rhythms by allowing the occurrence, during hours of natural darkness (both indoors and outdoors), of activities normally performed during daylight hours, such as food intake or social meetings. This has a profound impact on the immune-endocrine circadian (24h) timing mechanisms and the other endogenous rhythms that have evolved to ensure that human behavior is more efficient when synchronized with variations in light (for circadian ones) and with other environmental circumstances such as weather and seasons [4].

The consequences on metabolic dysfunctions and obesity of the de-synchronization of biological rhythms due to the effect of artificial light have been studied and verified [4], as well as the possible consequences for the risk of breast cancer and prostate cancer [5].

## 2 MELATONINE, (NEURO)STEROIDS AND HEALTH

Some studies suggested that sleep-wake cycle interruptions and artificial light pollution might be triggering factors in bipolar disorder [6-8]. Moreover, the role of sleep deprivation in inducing mania is well-known, although the mechanisms that underlie this action are unknown [9].

A recent review has confirmed that the suppression of melatonin incretion at night and the subsequent effect on DNA methylation play an important role in the development of prostate and breast cancer [10]. The role of melatonin dysregulation on the genesis of those diseases is intuitively understandable, given the interaction of melatonin with steroid hormones. Overall, although melatonin steroid-induced mechanisms are very complex, in general, they decrease estradiol and increase progesterone levels [11], so the block of melatonine at night, due to light pollution, unbalances the estradiol / progesterone ratio in favor of estradiol.

The same effects may also be relevant in bipolar disorder. Neurosteroids are synthesized in the brain and have effects on the neuroreceptors of many brain regions; moreover, peripheral synthesized steroids cross the encephalic barrier and produce effects similar to those of the neurosteroid in modulating brain excitability [12].

Laboratory and clinical findings show that progesterone derivate neurosteroids such as allopregnanolone and allotetrahydrodeoxycorticosterone influence mood and mood disorders [13, 14]. In rat hippocampus, cerebral cortex and serum, pregnenolone levels were increased by some atypical antipsychotics, such as clozapine and olanzapine, which possess an effective stabilizing action on bipolar disorder [15, 16]. In lithium-treated mice, the blood levels of allopregnanolone and pregnenolone were found higher than those of controls [17]. Women living with bipolar disorder frequently show an exacerbation of the disease during their menstrual cycle; the onset and recurrence of critical episodes of bipolar disorder in women are frequent after giving birth, just coinciding with the drop of neurosteroid derivatives of progesterone [13]. In the premenstrual phase, women who are recovering from an episode of bipolar disorder had a plasma concentration of allopregnanolone higher than in women putatively healthy or with major depressive disorder. In contrast, the blood level of allopregnanolone was found low during depressive episodes and the antidepressant fluoxetine was found to contrast this effect [18]. These findings seem to indicate that derivatives of progesterone have significant mood-stabilizing effect; accordingly pregnelonole was found effective and safe in the treatment of bipolar depression [19].

In conclusion, we advance the hypothesis that the blockade of night-time production of melatonin due to light pollution may play a role in the genesis of bipolar disorder also as a consequence of the effect that melatonin exerts on the equilibria of steroid hormones. However, in future research it will be necessary to take into account the complexity of this effect, which is gender-different, both for the different level and role of progesterone derivatives and the concomitant effects of melatonin on testosterone, which differ by gender.

If, as outlined by prospective studies [20], those with a depressive disorder (a disorder that has a 3/1 female-male ratio) are more at risk of experiencing bipolar disorder (a disorder that has a 1/1 male female ratio), the risk of bipolar disorder in males with a basic vulnerability to depressive disorders is amplified by disruption of sleep and biorhythms caused by life in modern cities.

## 3 TOWARDS AN EVOLUTIONARY VIEW OF BIPOLAR DISORDER

From an evolutionary perspective, it can be assumed that having an excess of energy during an awakening night episode may have had an adaptive effect. For a species accustomed to resting at night, a sudden awakening due to a sudden light (or noise) may certainly associate with an alarming condition (a fire? an enemy attack?) that requires consumption of extra resources. It can be imagined that if the city demands that biological rhythms be broken, people with a basic predisposition to living with biologic rhythms different from what was normal in a previous era may be in an adaptive state. The problem is that the current changed habits of awakening in the night and light pollution do not match the typical pattern of energy disposal with respect to the tasks fixed by millennia of evolution. We are facing an evolutionary decoupling of habits and adaptive demands.

The immense growth of urban areas taking place in the modern world can also be due to the fact that a stimulating milieu could offer opportunities to improve the life of people coming from deprivation or war. Thus, it could select people who are driven to novelty seeking and are explorers with hyperhythmic temperaments, which is also consistent with studies on migrants by will and not as refugees [21]. From this point of view, a crisis with imbalance of mood could be considered today the more common way to manifest psychological malaise for people which tries and fails to adapt to a new world. The psychopathological disturbance would be the deranged side of those who find it difficult to fit into the new world of 24/7 activities. But the new lifestyle also influences those who adapt and show behaviors, reactions and responses that might resemble the disorder, but are on the adaptive side.

If mood disorders shorten life expectancy and reduce the birthrate due to both lesser sexual activity in periods of depression and intake of drugs that inhibit sexual activity during both the depressive and manic phases, on a genetic basis this should result in a decrease in the prevalence of the disorder. However, many surveys have found that mood disorders and bipolar disorder in particular continue to increase in Western societies [22-24].

A possible explanation for this paradox may be that those who have so called “predisposition to mood disorders” but do not become ill will live better and reproduce in the new world of 24/7 activity.
